# Steady γ-Ray Effects on the Performance of PPP-BOTDA and TW-COTDR Fiber Sensing

**DOI:** 10.3390/s17020396

**Published:** 2017-02-17

**Authors:** Isabelle Planes, Sylvain Girard, Aziz Boukenter, Emmanuel Marin, Sylvie Delepine-Lesoille, Claude Marcandella, Youcef Ouerdane

**Affiliations:** 1 Univ Lyon, Laboratoire Hubert Curien (LabHC), Université de Lyon, CNRS UMR 5516, F-42000 Saint-Etienne, France; sylvain.girard@univ-st-etienne.fr (S.G.); aziz.boukenter@univ-st-etienne.fr (A.B.); emmanuel.marin@univ-st-etienne.fr (E.M.); ouerdane@univ-st-etienne.fr (Y.O.); 2 Agence Nationale Pour la gestion des Déchets Radioactifs (Andra), F-92298 Châtenay-Malabry, France; sylvie.lesoille@andra.fr; 3 CEA DAM DIF (French Alternative Energies and Atomic Energy Commission), F-91297 Arpajon, France; claude.marcandella@cea.fr

**Keywords:** PPP-BOTDA, TW-COTDR, gamma radiation, Brillouin scattering, Rayleigh scattering, optical fiber, optical sensors

## Abstract

We investigated the evolution of the performances of Pulse Pre Pump-Brillouin Time Domain Analysis (PPP-BOTDA) and Tunable Wavelength Coherent Optical Time Domain Reflectometry (TW-COTDR) fiber-based temperature and strain sensors when the sensing optical fiber is exposed to two γ-ray irradiation conditions: (i) at room temperature and a dose rate of 370 Gy(SiO_2_)/h up to a total ionizing dose (TID) of 56 kGy; (ii) at room temperature and a dose rate of 25 kGy(SiO_2_)/h up to a TID of 10 MGy. Two main different classes of single-mode optical fibers have been tested in situ, radiation-tolerant ones: fluorine-doped or nitrogen-doped core fibers, as well as Telecom-grade germanosilicate ones. Brillouin and Rayleigh Sensitivities of N-Doped fibers were not reported yet, and these characterizations pave the way for a novel and alternative sensing scheme. Moreover, in these harsh conditions, our results showed that the main parameter affecting the sensor sensitivity remains the Radiation Induced Attenuation (RIA) at its operation wavelength of 1550 nm. RIA limits the maximal sensing range but does not influence the measurement uncertainty. F-doped fiber is the most tolerant against RIA with induced losses below 8 dB/km after a 56 kGy accumulated dose whereas the excess losses of other fibers exceed 22 dB/km. Both Rayleigh and Brillouin signatures that are exploited by the PPP-BOTDA and the TW-COTDR remain unchanged (within our experimental uncertainties). The strain and temperature coefficients of the various fibers under test are not modified by radiations, at these dose/dose rate levels. Consequently, this enables the design of a robust strain and temperature sensing architecture for the monitoring of radioactive waste disposals.

## 1. Introduction

Optical fiber sensors (OFSs) are at the cutting edge of attractive technology for integration in severe environments, especially those associated with radiation constraints. Using OFSs, it is possible to monitor a large range of environmental parameters such as temperature, strain, pressure, hydrogen presence or radiation dose [1,2,3]. These components are considered as promising candidates for monitoring in the French deep geological repository for radioactive wastes, a project called Cigéo that will be associated with such challenging environmental constraints [4]. Cigéo is dedicated to High-Level (HL) and Intermediate-Level Long-Lived (ILLL) radioactive wastes. Structural health monitoring of repository cells will contribute to ensure long-term safety, operation security and reversibility of waste emplacement; moreover, sensing must be qualified for severe environmental constraints. Among the extreme expected conditions, two aspects can be noticed in the context of this paper: first, the temperature will vary from 30 °C to 90 °C; second, the radiation dose rate ranges from 1 to 10 Gy/h depending on the waste categories, leading to TID of about 10 MGy. This maximum TID is estimated with regards to the facility required lifetime (100 years). Under these conditions, the different possible applications for OFSs concern the monitoring of both strain and temperature changes within the radioactive waste repository cells liners [4].

More precisely, the French concept of cells, for ILLL wastes, consists of tunnels with concrete liners, whose dimensions are about 8 m in diameter and 600 m long. For HL waste cells, metallic liners are envisioned; the whole cell could reach 100 m, made of 2 m long tubes. There is a need to monitor the global evolution (1 km/200 m distance range for loop configuration) as well as local mechanical evolution of the facility, especially at the locations of transitions between pieces where the required spatial resolution increases up to ~10 cm. The gamma radiation dose rate depends on the waste category. In particular, the worst case for ILLL radioactive wastes, consists in a dose rate of 1 Gy(air)/h leading to TID up to 0.8 MGy in 100 years, whereas HL activity radioactive wastes are associated with higher dose rates, up to 10 Gy/h, leading to an accumulated dose of 8 MGy during the same period of one century.

The challenges in terms of performance concern first the spatial resolution of the measurements that implies consideration of distributed sensor architectures, and second, the radiation tolerance of the chosen sensors, and finally, the discrimination between the strain and temperature evolutions using the simplest and efficient detection scheme.

In the presence of this harsh environment, the use of radiation-hardened optical fibers is mandatory in order to provide distributed measurements of both temperature and applied strain along a sufficient fiber length. For the sensors exploiting the silica structure signature, it is also mandatory to consider the possible radiation-induced refractive-index changes that could originate from several origins: radiation-induced point defects and densification. Several sensor classes have already been recently tested under irradiation, such as those exploiting the fiber Brillouin [5,6,7,8,9], Rayleigh [3,10] and Raman [2] scattering signatures of silica-based fibers. Whereas the Raman technology detects only the temperature, the Brillouin and Rayleigh scatterings are sensitive to both the applied strain and the temperature. Radiation was shown to dramatically affect the temperature sensing response based on Raman scattering in a single-ended scheme, even if radiation-hard fiber is selected [2]. For this reason, some of Andra’s programs are now more focused on Brillouin and Rayleigh sensor technologies.

In this study, the performances of Brillouin and Rayleigh distributed sensors in a radiation environment are conducted on-line, meaning during the irradiation run. The vulnerability of three different single-mode fibers with acrylate coatings is tested: F-doped, N-doped and Ge-doped fibers. We investigated the radiation effects on two architectures of sensors: PPP-BOTDA and TW-COTDA. These two technologies sound promising for several reasons. First, at the system level, it is possible to design a unique sensor interrogator combining the two techniques and using single-mode fibers at 1550 nm, the part of the spectral domain where the RIA is limited with respect to UV-visible domain [5,7]. Second, in a more complicated scheme, it is possible to combine the two sensing techniques onto a unique fiber allowing discrimination, along the entire fiber length, between the respective evolutions of strain and temperature with high spatial resolution (up to 2 cm) and sensing distance (up to tens of km) [11,12,13]. If adapted to our targeted harsh conditions, this could overcome the observed issues in terms of implementation and event localizations. To the best of our knowledge, the on-line performances (during irradiation) of both the PPP-BOTDA and TW-COTDR sensing technologies are not yet documented in the literature. The γ-radiation effects on Brillouin responses were studied with BOTDA in [6], while the γ-radiation effects on Rayleigh were studied with OFDR technology in [10]. These BOTDA and OFDR technologies differ in terms of architectures from the ones discussed here, and the complex basic mechanisms of radiation effects on silica-based optical fibers can differently alter their sensing performances.

## 2. Distributed Sensing Measurements

The sensing optical fiber is probed by the Hybrid Brillouin-Rayleigh (HBR) system NBX-7020 (from Neubrex Technologies, Kobe, Japan) [11,12,13], selected for its large 6 dB dynamic range and its high spatial resolution (few centimeters). The main specification of this technology remains in the simultaneous characterizations of both Rayleigh and Brillouin signatures for the same single optical fiber. This is of utmost importance when practical implementation is considered. In our case and for our application, we initially developed sensing cables with several optical fiber types: multimode ones to perform temperature sensing distributed with Raman scattering systems and singlemode fibers for strain sensing based on Brillouin or Rayleigh scattering responses. However, even localization along these two different fibers, although collocated inside the same single cable, proved to be difficult (at the centimeter scale over a km range [14,15]). We also tested singlemode Raman devices, which proved to be highly sensitive to curvature [15]. This is why we turned towards Rayleigh and Brillouin association into a unique singlemode fiber.

### 2.1. Principle of PPP-BOTDA

The Brillouin scattering response is based on Pulse Pre Pump-Brillouin Time Domain Analysis (PPP-BOTDA) with a probe laser wavelength at 1550 ± 2 nm. For the BOTDA, the pre-pump technique includes two types of pulses: a pre-pump laser with a short pulse and a detection pump with a larger pulse. These different pulses improve the spatial resolution along the sensing fiber up to two centimeters, whereas it is in the order of 50 cm for the other Brillouin-related sensors. It is well known that the Brillouin Frequency Shift (BFS, Δυ_B_) evolution is linear versus both the applied strain and temperature. This can be expressed as:

Δυ_B_ = Cε_B_ × Δε + C_TB_ × ΔT (1)

where Cε_B_ and C_TB_ are the strain and temperature coefficients, respectively. These two coefficients depend on the sensing optical fiber characteristics: core/cladding compositions, nature of its coating (acrylate, polyimide, metal…), drawing conditions, packaging… For the as-drawn standard SMF28 fiber from CORNING, the typical values reported in the literature [11,12] are 0.05 MHz/µε for Cε_B_ and 1.07 MHz/°C for C_TB_.

### 2.2. Principle of TW-COTDR

The sensing fiber Rayleigh scattering signature is based on the Tunable Wavelength Coherent Optical Time Domain Reflectometry (TW-COTDR) scheme [12]. The operating tunable laser wavelength range is between 1530 and 1560 nm. This technique enables single-end access distributed measurement and provides a spatial resolution of two centimeters. Simultaneous measures of both strain and temperature changes along the fiber length are performed with respect to a reference measurement for all OFSs exploiting the Rayleigh fiber signature. The cross-correlation of Rayleigh scattering traces for the reference and at a given time t provides a frequency shift related to changes of the fiber temperature or/and strain applied to the waveguide. Indeed, the system supplies Rayleigh frequency shift Δυ_R_, which depends on the applied strain Δε and temperature changes ΔT through the TW-COTDR relation:

Δυ_R_ = Cε_R_ × Δε + C_TR_ × ΔT (2)

where Cε_R_ is the strain coefficient, and C_TR_ is the temperature coefficient of the fiber used as the sensing element. Typical values for the SMF28 fiber are 0.78 GHz/µε for Cε_R_ and 1.5 GHz/°C for C_TR_ [11,12]. For a bibliographic coherence effort with the referenced OFDR Equation (3), this TW-COTDR equation is divided by the mean frequency υ. The coefficients then become 0.78 µε^−1^ and 7.75 × 10^−6^ °C^−1^, respectively.

(3) $$\frac{{\Delta \upsilon }_{R}}{\upsilon_{R}}\;={\;C}_{\varepsilon R\prime }\times \Delta \varepsilon \;+{\;C}_{\mathrm{TR}\prime }\times \Delta T$$

## 3. Materials and Experimental Procedures

### 3.1. Tested Fibers

Three different single-mode optical fibers have been tested. All possess an acrylate coating whose maximum temperature of operation is about 80 °C. However, the samples differ by their composition that is known to strongly impact the fiber response [3,5]. Their exact core compositions were investigated by both Electron MicroProbe Analysis (EMPA) and Energy Dispersive X-ray (EDX) analysis, and the results are reported in Table 1. The SM-Ge was elaborated using the Modified Chemical Vapor Deposition (MCVD) process and contains a high Ge concentration (28 wt %) in its core and a pure-silica-cladding. Its Brillouin signature was shown to be very sensitive to high doses of radiations: UV or γ-rays [5,6,7]. The SM-F sample was designed with a slightly F-doped core and a higher F-concentration in the cladding (up to about 1.8 wt %). This type of MCVD F-doped fiber presents a low permanent RIA level in the infrared domain, even at high irradiation doses (up to 10 MGy). Our previous post-mortem studies, performed several weeks after the irradiation run reveal a limited room-temperature permanent Radiation-Induced Brillouin Frequency Shift (RIBFS) for this fiber class. RIBFS remains below 2.3 MHz up to a 10 MGy dose [5,6] whereas changes in its C_TR_ and Cε_R_ coefficients are below 5% after the same TID [10]. The SM-N fiber is obtained by a Surface Plasma Chemical Vapor Deposition (SPCVD) with an N-doped core and a pure silica cladding. It was previously shown that this fiber type presents low RIA too, especially during irradiation at low γ-ray dose and dose rate [16]. However, to the best of our knowledge, no data have been reported regarding the Brillouin or Rayleigh signatures of such N-doped fibers. Moreover, for radiation sensitive fibers such as Ge-doped ones, in-situ tests have been performed [5] and no “transient degradation” of the Brillouin response has been observed during low dose rate exposure up to total cumulative dose of 159 kGy.

### 3.2. Fiber Sample Packaging for Radiation Tests

For on-line tests, we used coils of each fiber with different packagings for PPP-BOTDA or TW-COTDR acquisitions and RIA measurements. Table 1 reports the SMF characteristics.

**Figure 1:**
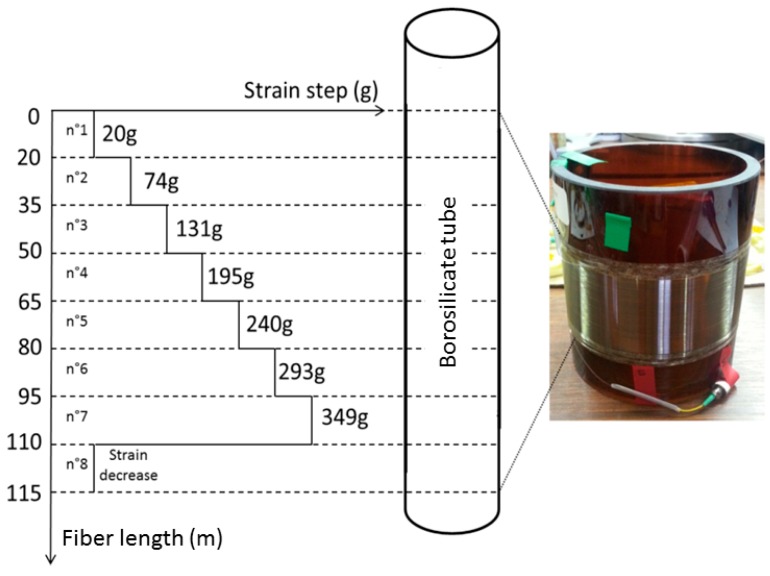
Schematic representation of the fiber coiling distribution with its associated strain steps. The Borosilicate holder darkened during the irradiation run.

To evaluate their sensing performances, a specific design was developed. Brillouin and Rayleigh responses are sensitive to both strain and temperature; then, to highlight radiation effects, it appears mandatory to precisely fix the strain applied to the fiber and to monitor the small temperature fluctuations during the irradiation run. If well done, it becomes possible to correct the rough results from these temperature changes to provide evidence for possible small radiation effects. This is done using the sample packaging illustrated in Figure 1 with a detailed scheme. The Fiber Under Test (FUT) is coiled around a borosilicate tube, by applying a controlled strain that is varied along the sample length, every 15 m, respecting the eight steps described in Table 2. Such a setup/design allows reduction of the uncertainty in the measurements of radiation effects and also permits in situ verification that radiation does not affect the fiber strain coefficient. Moreover, the RIA measurements were performed on stress-free samples with OTDR on both on-line and in post-mortem configurations.

### 3.3. Fiber Calibration Procedure

This coiling technique allows to determine the fiber strain dependence if its Young’s modulus is well known. For the specialty fiber types used in the test, this was not the case. That is why strain sensitivity was also measured with an additional and more standard technique.

**Figure 2:**
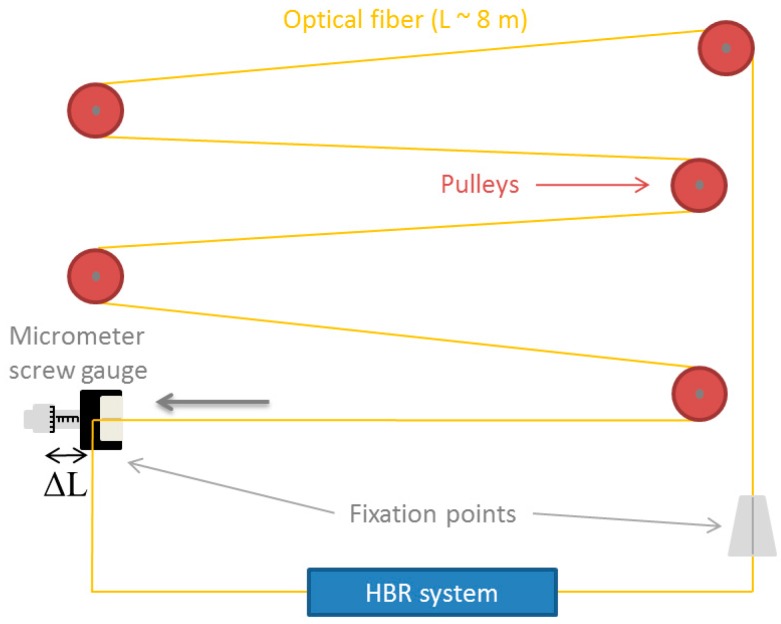
Schematic representation and picture of the strain calibration device.

Before the irradiation, the entire set of Cε_B_, Cε_R_, C_TB_ and C_TR_ coefficients was determined for the three studied fibers by using two dedicated setups developed at LabHC in France. For mechanical measures, a home-made strain calibration device was used to vary the strain applied to the as drawn fiber. The principle is based on a fiber supported by pulleys and fixed at the extremities, and strained over the section of length L by applying a stepwise displacement ΔL using a micrometer screw gauge (Figure 2), so the applied strain is given by: ε = ΔL/L. The total sample length was 8 m, while the strain step was 100 µε in our case.

In order to determine the temperature sensitivity coefficients, pristine fiber samples 10 m long were placed in an oven (Binder) equipped with a type K thermocouple. The oven temperature was varied between 30 °C and 80 °C with steps of 10 °C with respect to the acrylate coating temperature limitation. To withstand the application temperature of 90 °C, the acrylate coating would be changed to a polyimide one for the final application. The HBR measurements were performed after a stabilization time of more than half an hour at each temperature step.

### 3.4. Irradiation Conditions

Since the dose and dose rates are known to govern the kinetics and levels of radiation-induced changes in silica, we selected two different irradiation conditions to qualify the monitoring system for Cigéo.

**Figure 3:**
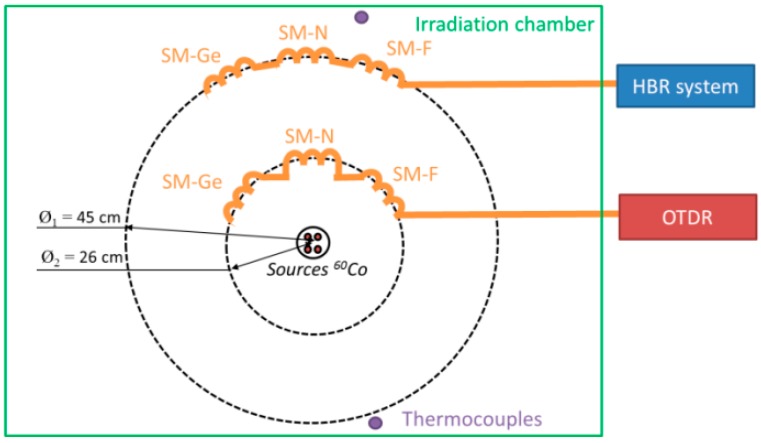
Schematic representation of the on-line experimental setup.

On-line tests were performed at the IRMA ^60^Co source of IRSN (Nuclear Safety and Radiation Protection Institute, Fontenay-aux-Roses, France) as this facility offers dose rates representative of the ones expected for the Cigéo project. The various dose rates were selected by changing the sample location (Figure 3) within the irradiation room. The run duration was about 150 h. In our case, we used two different dose rates. The lowest one, 370 Gy/h obtained at 45 cm far from the irradiation source, is associated with the samples devoted to the PPP-BOTDA and TW-COTDR measurements, leading to a final deposited dose of 56 kGy(SiO_2_), while the highest one, 1 kGy/h, is devoted to the Radiation Induced Attenuation (RIA) measurement at 1550 nm using the OTDR technique. In this case, the total deposited dose is about 150 kGy. All our FUTs were connected to radiation tolerant fiber pigtails (50 m length) for signal transport to the interrogators that are located in an instrumentation zone free of radiation. The irradiation room temperature was controlled by two thermocouples during the whole campaign and showed variations between 19 °C and 28 °C. The measurements were performed during (on line) and after (post-mortem) the entire irradiation run.

We also performed an accelerated aging test (at higher dose rates) in order to evaluate the possible impact of the total dose received by the sensors during the entire exploitation period of Cigéo. The irradiation facility for this post-mortem campaign was the BRIGITTE (Big Radius Installation under Gamma Irradiation for Tailoring and Testing Experiments) ^60^Co facility of SCK-CEN (Belgium). The maximal dose rate was 25 kGy/h, and the targeted maximal deposited dose was 10 MGy. The irradiation room temperature was controlled by a set of thermocouples during the whole campaign, and the reported values varied between 30 °C and 55 °C.

## 4. Experimental Results

### 4.1. Determination of Strain Coefficients before Irradiation

**Figure 4:**
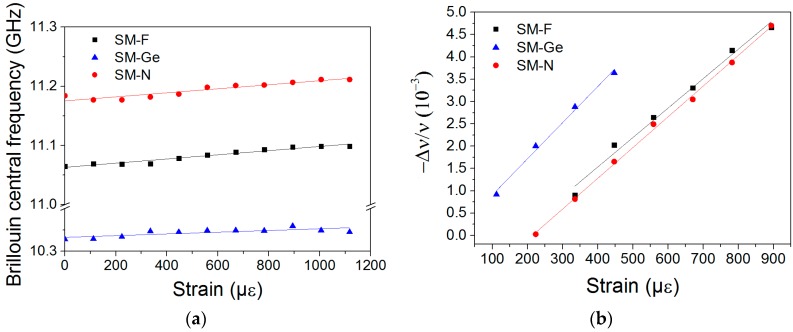
(**a**) Brillouin central frequency; and (**b**) Rayleigh relative spectral shift, as a function of the applied strain for SM-F (black square), SM-Ge (blue triangle) and SM-N (red circle) samples.

The Brillouin and Rayleigh strain coefficients were determined using the setup previously described by maintaining the temperature constant at 21 °C. The Brillouin peak frequency and its relative spectral shift versus the applied strain are presented in Figure 4a and b respectively. The strain sensitivity coefficient was evaluated using a linear regression fit procedure. The Brillouin strain coefficients of the tested fibers Cε_B_(SM-F), Cε_B_(SM-Ge) and Cε_B_(SM-N) are 0.035 MHz/µε, 0.017 MHz/µε and 0.034 MHz/µε, respectively. These coefficients are all lower than that reported in the literature for the SMF28 fiber: 0.05 MHz/µε [11,12]. The Rayleigh strain coefficients of the same set of samples are 0.07 µε^−1^ (SM-F), 0.08 µε^−1^ (SM-Ge) and 0.07 µε^−1^ (SM-N), respectively, comparable to the SMF28 strain coefficient: 0.78 µε^−1^ [10]. To the best of our knowledge, N-doped fibers have never been characterized for their sensing ability point of view, and the obtained results may enable new sensing scheme designs based on such fibers.

### 4.2. Determination of Temperature Coefficients before Irradiation

**Figure 5:**
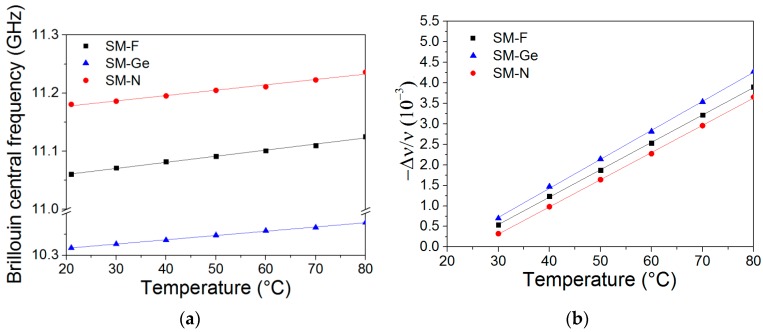
(**a**) Brillouin central frequency; (**b**) Rayleigh spectral shift as a function of temperature for the SM-F (back square), SM-Ge (blue triangle) and SM-N (red circle) samples.

The Brillouin and Rayleigh temperature coefficients were determined by the calibration procedure previously explained. Brillouin peak frequency and its spectral shift with a temperature increase are reported in Figure 5a,b, respectively. The Brillouin temperature coefficients (C_TB_) are 1.002 MHz/°C (SM-F), 1.042 MHz/°C (SM-Ge) and 0.893 MHz/°C (SM-N). These results are consistent with the values reported for the reference SMF28 fiber (1.07 MHz/°C) [11,12]. The Rayleigh temperature coefficients of the same tested fibers set C_TR’_(SM-F), C_TR’_(SM-Ge) and C_TR’_(SM-N) are 6.62 × 10^−6^ °C^−1^, 6.91 × 10^−6^ °C^−1^ and 6.56 × 10^−6^ °C^−1^, respectively, and they are also consistent with the SMF28 temperature sensitivity (7.75 × 10^−6^ °C^−1^) [11,12].

### 4.3. Steady γ-Ray RIA at 1550 nm

**Figure 6:**
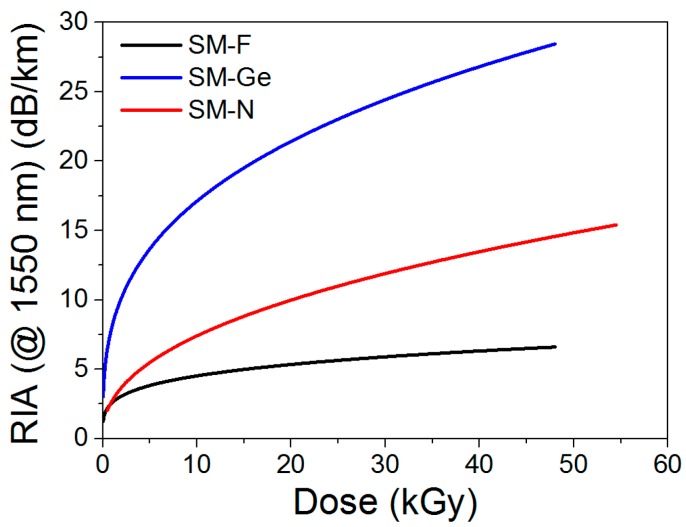
Radiation Induced Attenuation (RIA) of tested fibers at 1550 nm during on-line irradiation (1 kGy/h): SM-Ge (blue), SM-F (black) and SM-N (red).

Radiation induced attenuation (RIA) was measured to evaluate the excess of losses induced by radiation in the three tested fibers: SM-F, SM-Ge and SM-N. RIA induced by γ-rays at 1550 nm in the fiber samples was measured with an Optical Time Domain Reflectometer (OTDR) [17]: a pulse width of 10 ns and an analysis time of 180 s. As expected [18], the RIA (at 1550 nm) strongly depends on the nature of the core dopants. The SM-Ge fiber exhibits the highest losses of ~30 dB/km for an irradiation dose of ~45 kGy, while the loss level is about 14 dB/km for the N-doped fiber at the same cumulative dose. The lowest RIA level is observed in the F-doped fiber with 5.8 dB/km at the same irradiation dose and wavelength. These values agree with the literature [5], and 7.5 dB/km is reported for F-doped fiber. This result confirms the specific hardness property of the F-doped fiber. E.M. Dianov et al. [19] have shown a post-irradiation RIA of ~3.2 dB/km at 1550 nm and after 10 kGy in their N-doped fiber, a value close to the one for a pure-silica core fiber with RIA level of ~1.5 dB/km. Our RIA kinetics are reported in Figure 6 for the on-line irradiation run. In our case, we observed losses of 7.4 dB/km (SM-N) and 4.5 dB/km (SM-F) after a 10 kGy dose at 1550 nm. These results confirm that the N-doped fiber RIA is indeed closer to the F-doped fiber loss compared with the Ge-doped fiber loss. The more important difference in the RIA level between F-doped and N-doped fibers than in [19] is probably explained by the fact that metastable defects in N-doped fibers have a stronger contribution to the RIA at this wavelength during irradiation than observed after irradiation.

As a consequence, the F-doped fiber is clearly the best candidate for the monitoring of temperature and strain parameters in Cigeo applications.

We could estimate the impact of the RIA on the OFS performance in terms of sensing lengths. Our acquisition system has an optical budget of 10 dB for PPP-BOTDA and 7 dB for TW-COTDR. At an irradiation dose of 45 kGy, the RIA is ~30 dB/km for the Ge-doped fiber, ~14 dB/km for the N-doped fiber and ~5.8 dB/km for the F-doped fiber, which means that the maximal sensing range will be reduced to about ~300 m (SM-Ge), ~710 m (SM-N) and ~1720 m (SM-F) for PPP-BOTDA and ~233 m (SM-Ge), ~500 m (SM-N) and ~1206 m (SM-F) for TW-COTDR. If no problem associated with the sensing range has been observed for our 60-m long fiber samples, this sensing length reduction has to be considered from the application point of view.

### 4.4. On-Line γ-Ray Effects on the PPP-BOTDA Response

**Figure 7:**
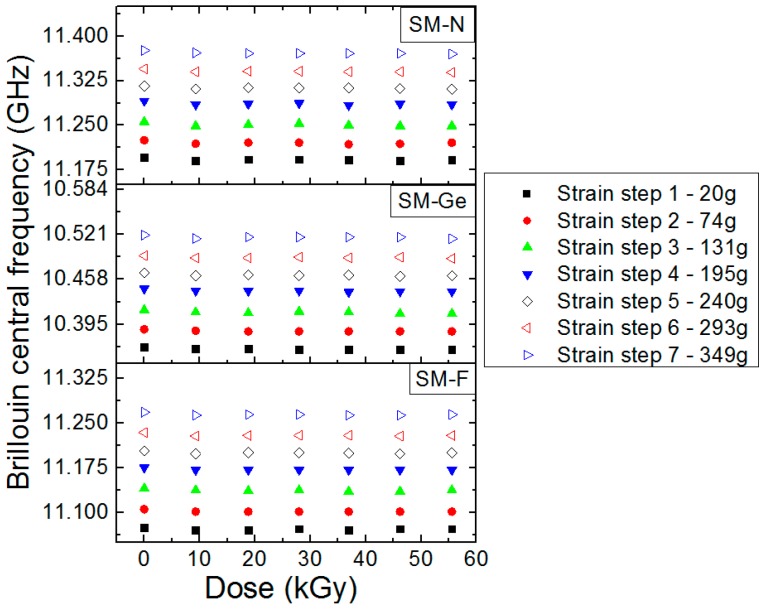
Evolution of the Brillouin central frequency as a function of the dose for SM-F, SM-Ge and SM-N fibers during the irradiation run at the dose rate of 370 Gy/h and different stain conditions in the various parts of the differently strained FUT.

The radiation effects on the Brillouin peak frequency for the three SM-F, SM-Ge and SM-N fibers are presented in Figure 7. Moreover, this evolution was also investigated at different strains. The error bars associated with the experimental measurements were obtained by considering the dispersion of the measured central frequency all along the 7-m length of each strained segment with a systematic error of 1 MHz attributed to our experimental setup. From the obtained results, it is obvious that the Brillouin central frequency remains mainly unchanged for all doses and all packaging conditions.

**Figure 8:**
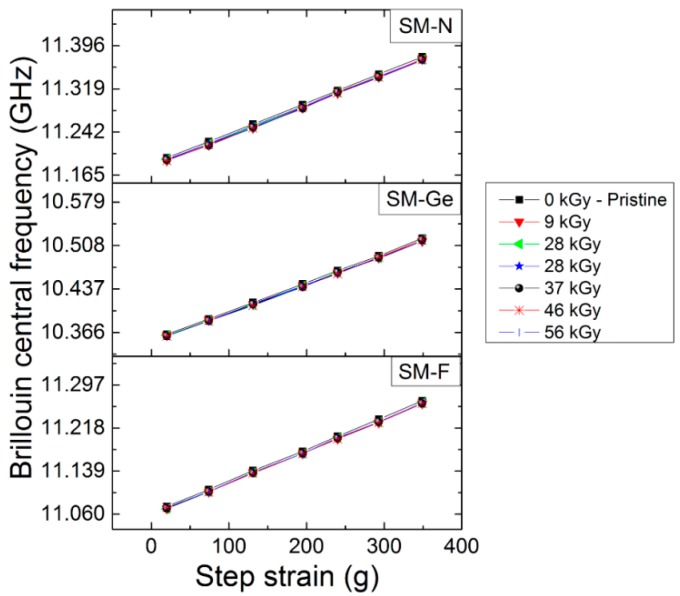
Evolution of Brillouin central frequency as a function of the applied stress for SM-F, SM-Ge and SM-N fibers, pristine and irradiated ones at different doses from 9 kGy to 56 kGy.

**Figure 9:**
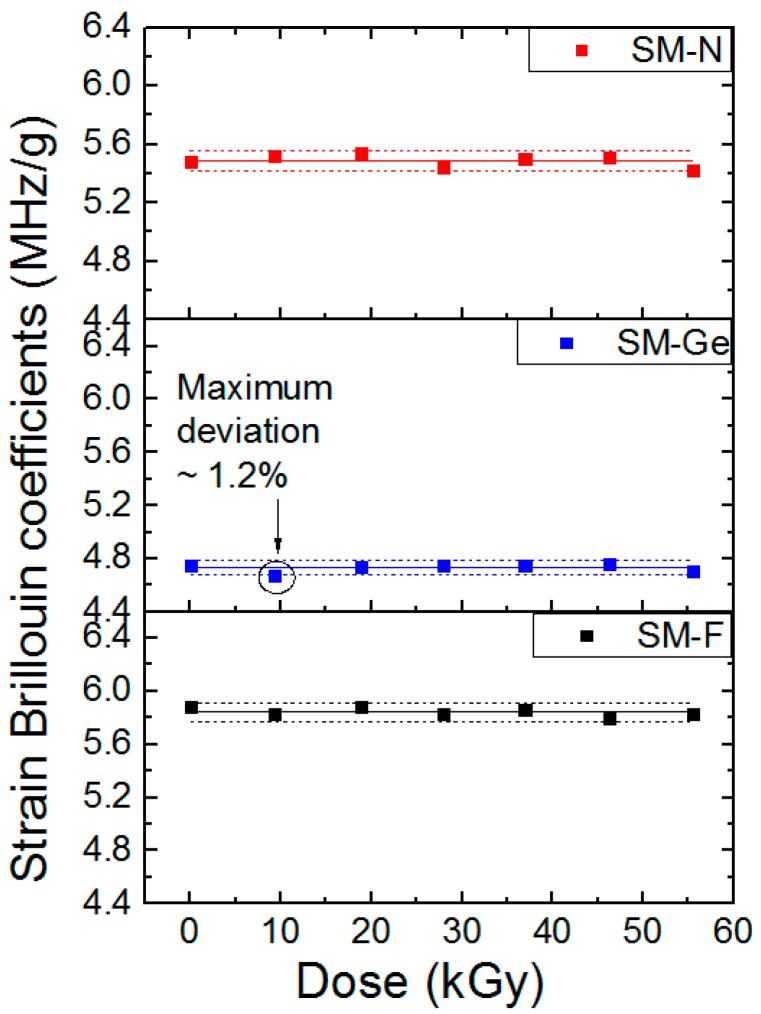
Strain Brillouin coefficients Cε_B_’ evolution as a function of dose for the F-doped fiber, Ge-doped fiber and N-doped fiber. The solid line in the middle indicates the average values while the dotted lines highlight a maximum variation of 1.2% for the Ge-doped fiber.

Thanks to our setup, it is possible to characterize online the spectral shift dependence with the strain step during the irradiation when the dose varies from 0 to 56 kGy. These dependencies are reported in Figure 8 for the three fibers and six different dose steps. For each dataset, a linear fit procedure was used for the fiber strain sensitivity calibration reported in Figure 9. During the irradiation, the variations of these coefficients were 0.7%, 1.2% and 0.9% for F-doped, Ge-doped and N-doped fibers, respectively. Such small changes are within the experimental uncertainties of our measurements.

### 4.5. On-line γ-Ray Effects on the TW-COTDR Response

**Figure 10:**
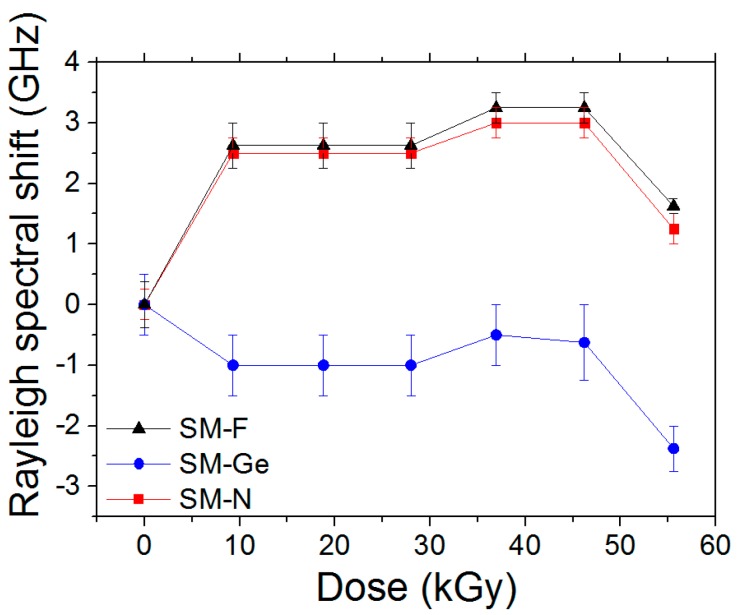
Rayleigh spectral shift as a function of dose, for the F-doped, Ge-doped and N-doped fibers.

The on-line γ-ray effects on the Rayleigh responses are presented for the same set of studied samples. As the Rayleigh reference signal was obtained after the fiber packaging, including the different strain steps, it is not possible to detect the various strain steps along the FUT using the TW-COTDR technique. As reported in Figure 10, the F-doped and N-doped samples show Rayleigh spectral shifts varying from 0 to 3.25 GHz and from 0 to −1 GHz for the Ge-doped fiber, without taking into account the point 56 kGy, which has a high dose. Most of the observed changes occur during the first 10 kGy, the Rayleigh shift being stable at higher doses. It is worth noticing that the Ge-fiber shows a negative Rayleigh shift, opposite to the N and F doped fibers.

### 4.6. Post-Mortem γ-Ray Effects on Temperature Coefficients

**Figure 11:**
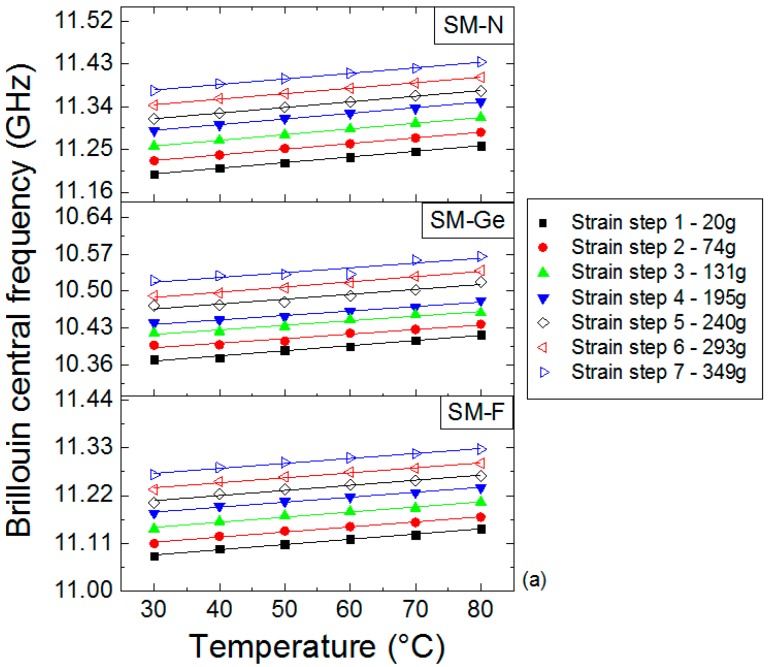

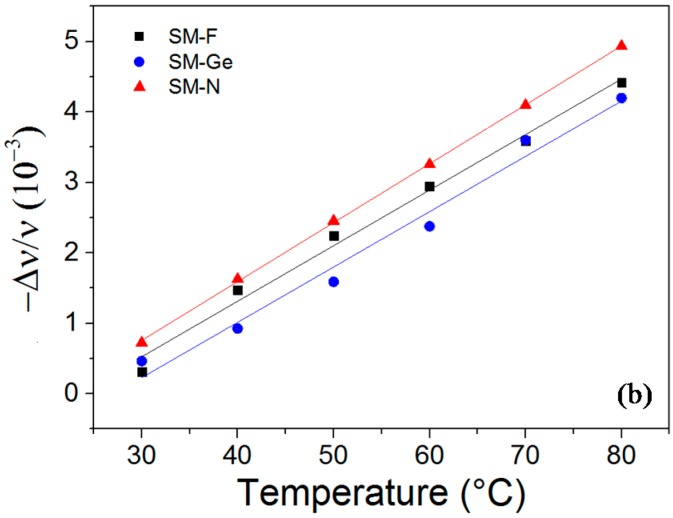
Brillouin central frequency (**a**) and Rayleigh relative spectral shift (**b**) evolutions as a function of temperature after irradiation at a dose of 56 kGy for the F-doped, Ge-doped and N-doped samples.

**Figure 12:**
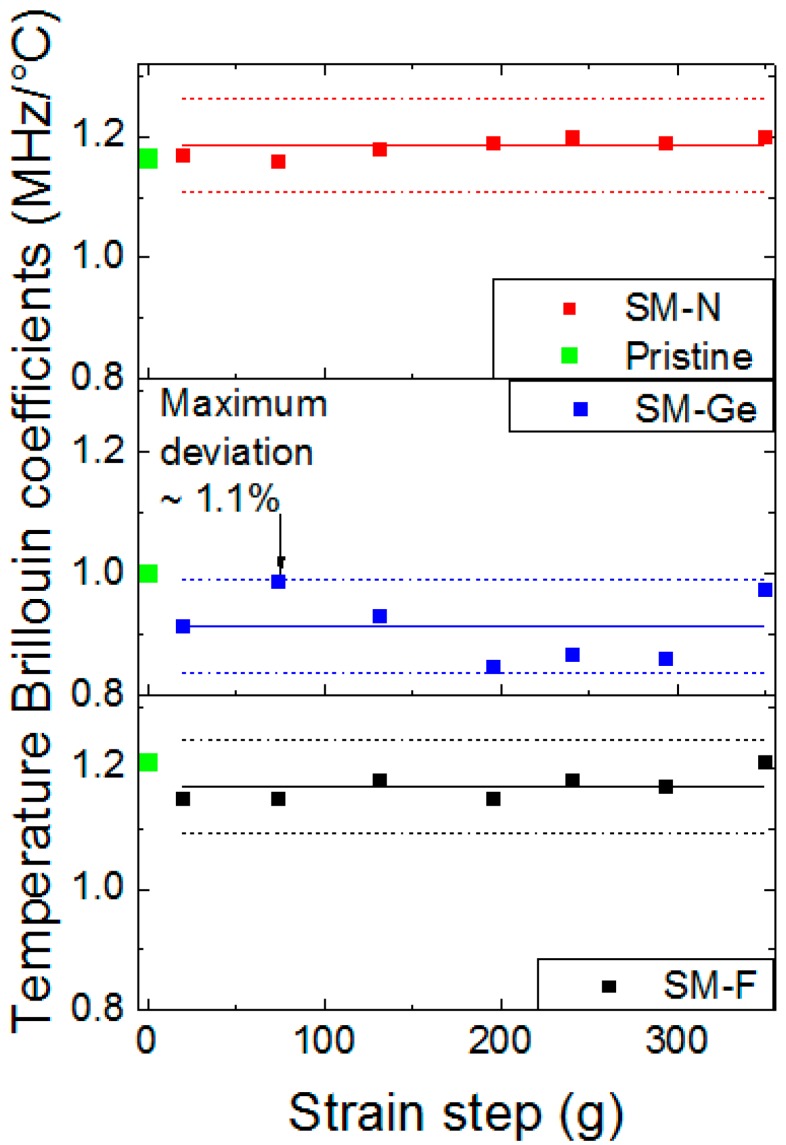
Brillouin Temperature coefficient C_TB_ evolution as a function of strain step for the F-doped, Ge-doped and N-doped fibers, after an irradiation dose of 56 kGy. The solid line in the middle indicates the average values while the dotted lines highlight a maximum variation of 1.1%. As a reference, green points are for pristine samples.

To determine Brillouin and Rayleigh temperature coefficients, we selected a range of length for each strained step of the irradiated sample characterized by a high signal to noise ratio in order to eliminate some temperature variation contributions that could misrepresent the final results. In each point of chosen range, we extracted the coefficient thanks to the linear fit (Figure 11). In this figure, the temperature error increases from 2.3 to 6 °C with the temperature (30 °C to 80 °C). All measurements of the spectral shift as a function of the temperature can be summarized in Figure 12, through the evolution of their temperature coefficients versus the irradiation dose for all the tested fibers. For each strain step of each sample (20, 74, 131, 195, 240, 293 and 349 g), Brillouin temperature coefficients Cε_T_ are independent of strain, even after irradiation at the maximal dose of 56 kGy. All the results are indeed scattered around their mean values (solid line in each graph) of 1.170 MHz/°C (SM-F), 0.913 MHz/°C (SM-Ge) and 1.184 MHz/°C (SM-N) within variations about 1% (Figure 12). The Brillouin temperature coefficients before irradiation and for loose fibers are 1.002 MHz/°C (SM-F), 1.042 MHz/°C (SM-Ge) and 0.893 MHz/°C (SM-N). For SM-F and SM-N fibers, the Brillouin temperature coefficients increase by 0.9% and 0.6%, respectively, with irradiation, but decrease for the SM-Ge sample. For Rayleigh measurements after irradiation, the strain steps are not visible because reference measurements were done after sample packaging. The Rayleigh temperature coefficients are 7.80 × 10^−6^ °C^−1^ (SM-F), 7.86 × 10^−6^ °C^−1^ (SM-Ge) and 8.44 × 10^−6^ °C^−1^ (SM-N). Their corresponding values before irradiation and for stress-free fibers are 6.62 × 10^−6^ °C^−1^ (SM-F), 6.91 × 10^−6^ °C^−1^ (SM-Ge) and 6.56 × 10^−6^ °C^−1^ (SM-N). We can notice that, for all fibers, the Rayleigh temperature coefficients increase between 0.9% and 1.3%. Such small variations are within the experimental uncertainties of our measurements and demonstrate that the Rayleigh temperature coefficients negligibly evolve with radiations at such dose levels. A complete view of all the coefficients with their relative errors is reported in Table 3.

### 4.7. Post-Mortem γ-Ray Effects on the PPP-BOTDA Response

**Figure 13:**
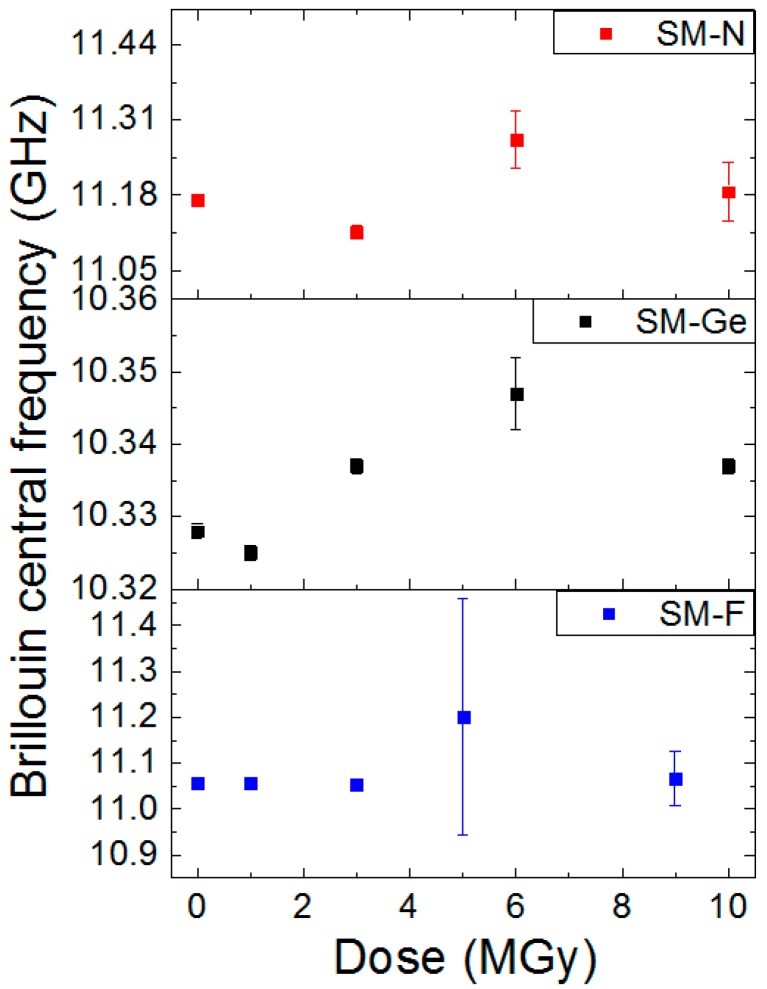
Evolution of the Brillouin central frequency as a function of the irradiation dose for the SM-F, SM-Ge and SM-N fibers. The experiments were done in post-mortem configuration (after the irradiation).

The post-mortem γ-ray effects on the PPP-BOTDA responses are presented in the Figure 13. We evaluated the PPP-BOTDA response for doses up to 10 MGy. For each fiber, the frequency increases with irradiation. Without taking into account the point 5–6 MGy which has an important uncertainty, the Brillouin frequency shifts for the SM-F, SM-Ge and SM-N samples are 3 MHz, 10 MHz and 20.5 MHz, respectively. These results are slightly superior to those previously presented in literature [6,9]. Brillouin scattering for SM-N appears very sensitive, i.e., 20 MHz and corresponds to an error of 22.4 °C.

## 5. Discussion

Three fibers, with different levels of strain applied, were characterized during on-line radiation exhibition, with Brillouin and Rayleigh scatterings. Brillouin measurements are summerized in Table 4, which compares the two strain coefficients before and after irradiation: The first coefficient Cε_B_ was determined using our mechanical setup described in Figure 2, while the second Cε_B_’ was evaluated using the various lengths differently strained on the sample coils. These coefficients decreased by 4–5 MHz/g, after an irradiation dose of 56 kGy, which can have a relative effect on the absolute measurments of the strain distibution all along the fiber length. This evolution of the coefficients introduces an error increasing up to 0.04 MHz/g.

## 6. Conclusions

Radiation effects on performances of Brillouin and Rayleigh scatterings have been studied for F-doped, Ge-doped and N-doped fibers. The Brillouin analysis shows that the Brillouin frequency and the Brillouin strain coefficient do not change with γ-ray irradiation doses up to 56 kGy. Furthermore, the Rayleigh spectral shift barely changes with the exposures, at least for the same dose levels. The F-doped and N-doped samples show Rayleigh spectral shifts varying from 2.63 to 3.25 GHz, whereas the Ge-doped fiber remains between −1 and −0.5 GHz. From the sensing performance point of view, this can be neglected. The influence of representative gamma radiation dose rates on Brillouin and Rayleigh fiber sensors was estimated, and the results are promising for the targeted application. At 1550 nm, the Ge-doped, N-doped and F-doped fibers exhibit excess losses of 22 dB/m, 7.4 dB/km and 4.5 dB/km, respectively. This is the first time that an N-doped fiber is studied for this application type, and it is not promising as the good sensitivity of these fibers is counterbalanced by a too high sensitivity of their scattering signatures to radiations. We also evaluated the Brillouin responses at the high dose levels associated with the long-term lifetime profile of the future Cigéo facility over the decades of monitoring. Our post-mortem measurements show that the fiber environment and the conditions for sample strain achievement can influence both the Brillouin and Rayleigh temperature coefficients. As a conclusion, all the radiation aspects of the temperature and strain monitoring by fiber sensors were encircled for the cigéo project. With the F-doped fiber, the design is validated for the continuation of this project. Based on the promising preliminary results reported in this paper, another irradiation campaign is already planned and devoted to the discrimination between the two parameters of interest using the two coupled measuring techniques in order to spatially discriminate the two parameters of interest: strain and temperature along a unique optical fiber.

## Figures and Tables

**Table 1:** Characteristics of tested fibers and tested samples.

Type	Core	Cladding	Coating
SM-F	F-SiO_2_ (0.2 wt %)	F-SiO_2_ (1.8 wt %)	Acrylate
SM-Ge	Ge-SiO_2_ (28 wt %)	Pure silica	Acrylate
SM-N	N-SiO_2_	Pure silica	Acrylate

**Table 2:** Strain step.

N°Step	1	2	3	4	5	6	7	8
Length (m)	0 to 20	20 to 35	35 to 50	50 to 65	65 to 80	80 to 96	95 to 110	110 to 115
Strain step (g)	20	74	131	195	240	293	349	Strain decrease

**Table 3:** Temperature coefficients extracted from Brillouin and Rayleigh measurements and relative errors after irradiation at TID of 56 kGy.

C_TB_ (MHz/°C)								C_TR‘_ (10^−6^ C^−1^)
N°Step	1	2	3	4	5	6	7	/
Strain step (g)	20	74	131	195	240	293	349	/
SM-F	1.15 ± 0.06	1.15 ± 0.07	1.18 ± 0.07	1.15 ± 0.05	1.18 ± 0.07	1.17 ± 0.06	1.21 ± 0.05	7.80 ± 0.04
SM-Ge	0.91 ± 0.18	0.99 ± 0.05	0.93 ± 0.13	0.85 ± 0.06	0.87 ± 0.07	0.86 ± 0.09	0.87 ± 0.06	7.86 ± 0.05
SM-N	1.17 ± 0.02	1.16 ± 0.02	1.18 ± 0.02	1.19 ± 0.02	1.20 ± 0.00	1.19 ± 0.02	1.20 ± 0.03	8.44 ± 0.06

**Table 4:** Strain Brillouin coefficients in the unit of MHz/µε and MHz/g.

Fibers	Cε_B_ before Irradiation (MHz/µε)	Cε_B_’ before Irradiation (MHz/g)	Cε_B_’ after Irradiation to 56 kGy (MHz/g)
F-doped fiber	0.035 ± 0.015	5.88 ± 0.02	5.83 ± 0.06
Ge-doped fiber	0.017 ± 0.039	4.75 ± 0.02	4.71 ± 0.06
N-doped fiber	0.034 ± 0.016	5.48 ± 0.02	5.42 ± 0.05
